# Adaptive Expression of MicroRNA-125a in Adipose Tissue in Response to Obesity in Mice and Men

**DOI:** 10.1371/journal.pone.0091375

**Published:** 2014-03-27

**Authors:** Malika R. Diawara, Christophe Hue, Steven P. Wilder, Nicolas Venteclef, Judith Aron-Wisnewsky, James Scott, Karine Clément, Dominique Gauguier, Sophie Calderari

**Affiliations:** 1 Institut National de la Santé et de la Recherche Médicale (INSERM) UMRS 872, Cordeliers Research Center, University Pierre & Marie Curie, Paris, France; 2 Institute of Cardiometabolism & Nutrition, ICAN, Pitié-Salpêtrière Hospital, University Pierre & Marie-Curie, Paris, France; 3 Wellcome Trust Centre for Human Genetics, University of Oxford, Oxford, United Kingdom; 4 National Heart and Lung Institute, Imperial College London, London, United Kingdom; University of Padova, Italy

## Abstract

MicroRNAs are emerging as new mediators in the regulation of adipose tissue biology and the development of obesity. An important role of microRNA-125a has been suggested in the pathogenesis of insulin resistance (IR). Here, we characterized the function of microRNA-125a in adipose tissue in a context of experimentally-induced IR and obesity in mice and in obese patients. We showed time dependent overexpression of the microRNA in adipose tissue of BALB/c and C57BL/6J mice in response to high fat diet (HFD) feeding. MicroRNA-125a expression was downregulated *in vitro* in insulin resistant 3T3-L1 adipocytes and *ex vivo* in adipose tissue of obese patients. *In vitro* modulation of microRNA-125a expression in 3T3-L1 adipocytes did not affect glucose uptake. Gene set enrichment analysis (GSEA) identified significantly altered expression patterns of predicted microRNA-125a gene targets in transcriptomic datasets of adipose tissue from HFD-fed mice and obese patients. Among genes that contributed to global enrichment of altered expression of microRNA-125a targets, Thyrotroph embryonic factor (*Tef*), Mannan-binding lectin serine peptidase 1, Reticulon 2 and Ubiquitin-conjugating enzyme E2L3 were significantly differentially expressed in adipose tissue in these groups. We showed that *Tef* expression is reduced in adipose tissue of obese patients following gastric bypass surgery. Our findings indicate that microRNA-125a expression in adipose tissue adapts to IR and may play a role in the development of obesity in mice and obese subjects through uncoupled regulation of the expression of microRNA-125a and its targets.

## Introduction

Obesity and type 2 diabetes mellitus (T2DM) are major health issues caused by a complex interplay between genetic risk factors and environmental influences, including sedentary lifestyle and change in feeding behavior [1], [2]. Insulin resistance (IR) is a cardinal pathophysiological feature in these chronic diseases [3]. Defining molecular signatures of the onset and progression of T2DM and obesity has become crucial to provide a deeper understanding of the mechanisms involved and uncover predictive and therapeutical targets. Owing to the complexity of molecular studies in humans and limitations in access to organ biopsies in patients and controls, animal models of obesity and T2DM represent powerful tools to identify etiological mechanisms of IR. Models of spontaneously-occurring T2DM, such as the Goto Kakizaki (GK) rat [4], and experimentally-induced obesity by high fat diet (HFD) feeding [5] are widely used to identify novel genes and gene pathways involved in IR. We have previously demonstrated contrasting pathophysiological responses to HFD in C57BL/6J and BALB/c mice [6], [7].

Progress in molecular genetic technologies, which allows deeper analyses of genome expression patterns contributing to, or reactive to, a disease phenotype, has underlined new molecular mechanisms mediated by microRNAs (miRs) in the biology of adipose tissue and the development of obesity [8], [9], [10], [11]. MiR is a family of highly conserved, single-stranded, 19–23 nucleotide long, non-coding, endogenous RNAs, which negatively regulate gene expression either by inhibiting translation or by degrading target mRNAs [12]. One miR can target several mRNAs and one transcript can be targeted by several miRs. Such interactions regulate various aspects of metabolism through pancreatic development, insulin biosynthesis, secretion and signaling, adipocyte differentiation and glucose uptake [8], [13].

We have demonstrated differential expression of a series of miRs in the GK rat model of polygenic IR [14], [15]. Consistent overexpression of miR-125a in both liver and adipose tissue of the GK rat suggested that this miR may play an important role in IR pathogenesis [14]. To further characterize its function in a context of experimentally-induced IR, we investigated the regulation of the expression of this miR and its proposed transcript targets in adipose tissue from C57BL/6J and BALB/c mice fed HFD and from obese patients. Our results indicate that miR-125a expression is reactive to IR and suggest that uncoupled differential expression of miR-125a and its mRNA targets may play a role in the development of obesity.

## Methods

### Patients

Seventeen severely obese patients, who were candidates for bariatric surgery and 7 non-obese subjects were included in a study protocol approved by the Ethics Committee of Hotel-Dieu Hospital, and signed informed written consent. Physiological parameters are listed in Table 1. In the group of 17 severely obese patients, 7 patients had T2DM. Body mass index was calculated from the measured body weight and height. Subcutaneous periumbilical adipose and omental biopsies were collected at the time of surgery. Blood samples were obtained at each time point after 12 h overnight fasting and stored at −20°C until assays of plasma total cholesterol, triglycerides, insulin, glucose, leptin, adiponectin, and liver marker, γ-glutamyltransferase (γGT). Pangenomic expression of subcutaneous adipose tissue was conducted before and after gastric surgery as described [16]. Fat and fat-free mass were determined by dual-energy X-ray absorptiometry (GE Lunar Prodigy Corp., Madison, WI). QUICKI was determined by a mathematical calculation using fasting glucose and insulin as described previously by Katz et al. [17]. These evaluations were performed before surgery and during the post-surgery follow-up. Plasma glucose, triglycerides, and total cholesterol levels were measured enzymatically. Serum insulin concentrations were determined with immunoradiometric assay (Bi-INSULIN IRMA CisBio International, Gif-sur-Yvette, France). Serum leptin and adiponectin were determined using radioimmunoassay kits (Linco Research, Saint Louis, MI, USA).

**Table 1:** Patient physiological parameters (F, Female; M, Male; BMI, Body mass index; TGL, Triglyceride; γGT, γ-glutamyltransferase; IL6, Interleukin 6; ApoB, Apolipoprotein B;QUICKI, Quantitative insulin sensitivity index).

Sex	Diabetic	Anti-diabetic treatment	Obese	Surgery age (years)	Body wt (kg)	BMI (kg.m^-2^)	Fasting glycemia (mM)	Fasting insulinemia (μU/ml)	HbA1C (%)	Fat mass (kg)	Fat free mass (kg)	TGL (mmol/l)	γGT	Leptin (ng/ml)	Adipo-nectin (μg/ml)	IL6 (pg/ml)	Apo B (g/l)	Total cholesterol (mmol/l)	QUICKI	Systolic blood pressure (mmHg)	Diastolic blood pressure (mmHg)
F	No	-	No	41	72	26,4	5,4	-	-	-	-	-	-	-	-	-	-	-	-	-	-
M	No	-	No	67	84	27,4	4	-	-	-	-	-	-	-	-	-	-	-	-	-	-
F	No	-	No	64	78	28,0	-	-	-	-	-	-	-	-	-	-	-	-	-	-	-
M	No	-	No	67	84	26,2	4,5	-	-	-	-	-	-	-	-	-	-	-	-	-	-
F	No	-	No	49	63	25,3	5,4	-	-	-	-	-	-	-	-	-	-	-	-	-	-
F	No	-	No	37	65	23,8	-	-	-	-	-	-	-	-	-	-	-	-	-	-	-
M	No	-	No	17	85	26,2	-	-	-	-	-	-	-	-	-	-	-	-	-	-	-
F	No	-	Yes	38	104	42,6	5,2	6,9	5,5	51	50	1	79	68	6,1	3,4	1	4,8	0,64	109	53
F	No	-	Yes	38	133	47	4,9	21,8	5,2	65	63	1,9	20	82	2,5	3	1	5,6	0,49	120	80
F	No	-	Yes	35	165	68,6	5,1	26,9	6,3	92	63	1,4	24	186	4	11,8	-	4,4	0,47	120	70
F	No	-	Yes	37	140	51	5	11,4	5,8	70	65	1,5	18	64	5,2	4,3	1	4,9	0,57	130	75
F	No	-	Yes	44	128	42,3	5,7	13,3	5,8	62	63	0,7	27	78	9	5,3	0,8	4,4	0,53	105	65
F	No	-	Yes	28	110	43	4,8	26,2	5,8	52	55	0,9	37	57	2,7	2,5	0,7	4,1	0,48	118	55
F	No	-	Yes	62	166	58,8	4,5	10,6	5,4	92	70	1,2	46	133	10,5	3,1	0,9	5,4	0,60	110	70
F	No	-	Yes	20	130	48,4	4,2	19,1	5,2	64	60	0,8	22	133	4,7	8,1	0,8	4,2	0,53	-	-
F	No	-	Yes	48	104	41,1	4,4	14,7	5,2	49	51	1,4	28	51	2,8	1,6	0,8	4,5	0,55	139	83
M	Yes	Yes	Yes	65	123	45,2	6,7	22,2	6,3	60	58	1	25	55	3,9	6,6	0,8	4,1	0,46	160	
M	Yes	No	Yes	49	120	36,9	6,2	28,7	6,2	42	73	1,3	119	24	4,2	2,5	0,7	3,9	0,44	120	70
M	Yes	Yes	Yes	31	167	47,2	10,8	31,2	9,1	81	80	1,2	81	52	1,9	4	1	4,3	0,40	130	90
M	Yes	Yes	Yes	54	192	64,2	6,6	17,1	6,2	-	-	2,8	169	70	2,6	6	1	4,9	0,49	100	60
F	Yes	Yes	Yes	46	113	41,3	4,6	10,7	6,1	51	57	1,1	38	45	4,3	1	1,2	5,4	0,59	120	80
F	Yes	No	Yes	61	111	41,6	6,5	32	6,7	55	51,2	1,7	35	51	5,6	2,2	-	3,5	0,43	133	72
F	Yes	Yes	Yes	29	164	60,8	6,8	23,9	8,5	84	68,5	3,1	-	54	4	6,2	0,2	4,7	0,45	145	95

### Animals

Male C57BL/6J and BALB/c mice were bred from Jackson laboratory stocks. Mice were weaned at 3 weeks and fed a control carbohydrate diet (CHD) containing 5% fat, 19% protein and 3.5% fibers (S&K Universal Ltd, Hull, United Kingdom). At 5 weeks, a group of mice was transferred to 40% HFD (High-fat diet) containing 40% saturated fat, 19% protein, 21% glucose (Special Diet Services, Witham, United Kingdom) *ad libitum*, whereas the control group remained on CHD. Mice fed HFD for 1 week and 15 weeks, and age-matched controls were killed by CO_2_ asphyxiation. Epididymal fat pads (EPD) were rapidly dissected and snap frozen in liquid nitrogen and then stored at −80°C. Animal procedures were carried out under United Kingdom Home Office personal and project licences and approved by the ethical review panel of the University of Oxford.

### Glucose tolerance and insulin secretion tests in mice

Intraperitoneal glucose tolerance tests were performed in overnight fasted mice. Briefly, mice were anesthetized with an intraperitoneal injection of sodium pentobarbital (Sagatal, Rhône Mérieux, Harlow, United Kingdom) and a solution of glucose (2 g/kg body weight) was injected intraperitoneally. Blood samples were collected from the tail vein before the injection and 15, 30 and 75 min afterward. Blood glucose was immediately determined with a glucose meter (Accucheck, Roche Diagnostics, Burgess Hill, United Kingdom). Plasma samples were stored at −80°C until assayed for immunoreactive insulin (ELISA, Mercodia, Uppsala, Sweden). Cumulative glycemia and insulinemia were calculated as the increment of the values of plasma glucose and insulin, respectively, during the test. Characterization of the short and long-term consequences of fat feeding on the onset and progression of insulin resistance, diabetes and obesity in C57BL/6J and BALB/c is described in Table 2.

**Table 2:** Phenotype characterization of the effects of 1 and 15 weeks of HFD feeding in C57BL/6J and BALB/c mice (CHD, Carbohydrate diet; HFD, High-fat diet).

	C57BL/6J 1week CHD	C57BL/6J 1week HFD	C57BL/6J 15weeks CHD	C57BL/6J 15weeks HFD	BALB/c 1week CHD	BALB/c 1week HFD	BALB/c 15weeks CHD	BALB/c 15weeks HFD
Body weight (g)	20.11±0.89(n = 8)	19.43±0.91 (n = 8)	33.06±0.77 (n = 8)	40.03±1.16 (n = 7)***	19.66±0.36 (n = 8)	20.50±0.38 (n = 8)	28.80±1.10 (n = 5)	28.98±1.42 (n = 5)
Body mass index (g.cm^2^)	2.44±0.03 (n = 8)	2.57±0.02 (n = 8)*	3.17±0.04 (n = 60)	3.94±0.06 (n = 35)*	2.38±0.05 (n = 8)	2.58±0.05 (n = 8)*	2.73±0.11 (n = 5)	2.94±0.09 (n = 5)
Epididymal fat pads weight (g)	0.16±0.01 (n = 8)	0.28±0.04 (n = 8)**	0.35±0.07 (n = 4)	1.14±0.19 (n = 4)**	0.34±0.04 (n = 8)	0.53±0.05 (n = 8)*	0.35±0.06 (n = 4)	0.69±0.04 (n = 4)**
Fasting Glycemia (mM)	5.23±0.24 (n = 8)	5.73±0.56 (n = 8)	6.05±0.34 (n = 8)	10.31±0.34 (n = 8)***	4.69±0.34 (n = 8)	4.73±0.25 (n = 8)	5.74±0.39 (n = 5)	6.80±0.49 (n = 5)
Cumulative glycemia (mM)	930.8±18.7 (n = 8)	1039.6±41.4 (n = 8)*	1193.1±33.8 (n = 8)	1622.7±66.9 (n = 8)***	867.0±54.3 (n = 8)	812.4±77.1 (n = 8)	1107.2±73.6 (n = 5)	1084.9±46.7 (n = 5)
Cumulative insulinemia (pM)	53.3±4.9 (n = 8)	47.5±6.6 (n = 8)	370. 9±78.1 (n = 4)	264.0±28.3 (n = 8)	35.4±1.5 (n = 8)	38.0±4.3 (n = 8)	41.2±2.7 (n = 5)	329.7±55.8 (n = 5)***

*p<0.05, **p<0.01, ***p<0.001 significant differences between mice fed with HFD and CHD.

### *In vitro* adipocyte differentiation from 3T3-L1 cells and induction of IR

3T3-L1 fibroblasts were differentiated into adipocytes by a protocol adapted from Nugent et al. [18]. 3T3-L1 fibroblasts (LGC standard, Molsheim, France), between passages 4 and 8 were cultured in a growing medium containing DMEM (Life technologies, Saint Aubin, France), 25 mmol/l glucose, 4 mmol/l L-glutamine, 1 mmol/l sodium pyruvate, 10% FBS (PAA laboratories, Velizy-Villacoublay, France), 1% penicillin/streptomycin in humidified air at 37°C and 5% CO_2_. Two days after confluence, cells were incubated in a differentiating medium containing the growing medium supplemented with 0.25 μmol/l dexamethasone, 500 μmol/l 3-isobutyl-1-methylxanthine (IBMX), 0.4 μmol/l insulin (Sigma Aldrich, Saint Quentin Fallavier, France). After three days, cells were placed in differentiating medium supplemented with 0.4 μmol/l insulin and at day7 mature adipocytes were obtained and maintained in differentiating medium with 5% FBS. After differentiation, 3T3-L1 adipocytes were weaned for two days in a low-glucose-medium (DMEM, 5.5 mmol/l glucose, L-glutamine, sodium pyruvate, 5% FBS). Then, IR was induced in 3T3-L1 adipocytes within 48 h following culture in a high-glucose/high-insulin-medium as described previously [19], [20]. IR was determined by glucose uptake assay. 3T3-L1 adipocytes were differentiated in 24-well plates and serum starved for 4 h in DMEM. They were then washed with a pre-warmed buffer containing PBS (Phosphate Buffered Saline), 0.5 mmol/l MgCl2, 0.9 mmol/l CaCl2, 0.2% BSA. Insulin (100 nmol/l) stimulation was performed for 20 min. Cells were then incubated with ^3^H-2-deoxy-glucose (18.5 kBq/well) for 10 min. Cells were solubilized and the radioactivity counted

### Modulation of miR-125a expression *in vitro* in 3T3-L1 adipocytes

After differentiation 3T3-L1 adipocytes were transfected during 48 h with either the mature miriDIAN mimic mmu-miR-125a-5p, cel-miR-67 as a transfection control, or 50 nmol/l mature miriDIAN inhibitor (Thermo Scientific, Dharmacon, Saint Leon-Rot, Germany) miR expression was assessed by q (quantitative) RT-PCR. Mmu-miR-125a-5p mature sequence targeted by miriDIAN inhibitor and corresponding to miriDIAN mimic is UCCCUGAGACCCUUUAACCUGUGA, cel-miR-67 mature sequence is UCACAACCUCCUAGAAAGAGUAGA. 3T3-L1 adipocytes were transfected in a total volume of 500 μl/well. Dharmafect1 (0.25 μl/cm^2^, Thermo Scientific, Dharmacon, Saint Leon-Rot, Germany) was used as a transfection reagent.

### MiR and gene expression

Tissues and cells were homogenized in Qiazol (Qiagen, Courtaboeuf, France). Total RNA was prepared as previously described [6]. The fraction containing miRs was extracted with miRNeasy mini kit (Qiagen, Courtaboeuf, France) and stored at −80°C. RNA concentration was determined with Nanodrop (Nanodrop ND-1000, Thermoscientific, Horsham, UK). QRT-PCR was carried out using either TaqMan microRNA assay (hsa-miR-125a-5p-002198, Life technologies, Saint Aubin, France) or SYBR Green expression assay (miScript reverse transcription kit, miScript SYBR Green PCR Kit, miScript Primer Asssay Mm_miR-125a_1-MS00001533, Rotor-Gene SYBR Green PCR Kit, Qiagen, Courtaboeuf, France). MiR-16-MS00031493, U6-MS00033740, (Qiagen, Courtaboeuf, France) and small nucleolar RNA202-001232 (Life technologies, Saint Aubin, France) were used as normalizing endogenous control for human tissues, cell culture samples and mouse tissues, respectively. Oligonucleotide sequences used are the following: Mouse *Rtn2* (*Reticulon 2*) 5′-CTTTAGCATCGTGTCCGT (forward), 5′-TGCAGCACTTTGCGGTAA (reverse); Mouse *Tef* (*Thyrotroph embryonic factor*) 5′-ATGGACCTGGATGAGTTC (forward), 5′-GCAGACTCCTTTCCTTCAA (reverse); Mouse *Ube2l3* (*Ubiquitin-conjugating enzyme E2L 3*) 5′-CCGCAAATGTGGAATGAA (forward), 5′-AGGAACAATAAGCCCTTG (reverse); Mouse *Masp1* (*Mannan-binding lectin serine peptidase 1*) 5′-AAAGATGCTGTGCTGGT (forward), 5′-GAATAGACTCCATAGCGAT (reverse); Mouse β-*Actin* 5′-GACGATGCTCCCCGGGCTGTATTC (forward), 5′-TCTCTTGCTCTGGGCCTCGTCACC (reverse); Human *Rtn2* 5′-AGCAGACGGAACGTTTGT (forward), 5′-GAATCCACGAGGTCTTCT (reverse); Human *Tef* 5′-ATGGACCTGGATGAGTTC (forward), 5′-CAGACTCCTTCCCTTCTA (reverse); Human *Ube2l3* 5′-AGGAGGCTGATGAAGGAC (forward), 5′-TCGGTGGTTTGAATGGGTA (reverse); Human *Masp1* 5′-ACAACGACAACAGGACCT (forward), 5′-GGTAAGGGTTTGGGAAGT (reverse); Human β-*Actin* 5′-CTCTTCCAGCCTTCCTTCCT (forward), 5′-AGCACTGTGTTGGCGTACAG (reverse).

### Genome-wide gene transcription profiling

Mice fat fed for 1 week (BALB/c) and 15 weeks (C57BL/6J) and their respective age matched controls, which were used for miR-125a expression analysis, were selected for gene transcription profiling. RNA probes prepared from BALB/c mice were hybridized to Affymetrix expression arrays 430 A and B (Affymetrix UK ltd, High Wycombe, UK), containing 22,690 and 22,576 probesets, respectively, and allowing quantification of the abundance of transcripts corresponding to 13,250 (chip A) and 7577 (chip B) independent gene and EST sequences. Probes prepared from C57BL/6J mice were hybridized to Affymetrix arrays U430 2.0, which were designed to contain all probesets of arrays 430 A and B on a single chip. Experiments are MIAME compliant and full protocols and data are publicly available (www.ebi.ac.uk/arrayexpress/) under the accessions E-BAIR-12 (BALB/c) and E-BAIR-9 (C57BL/6J). Affymetrix-based gene transcription profiling was performed as previously described [6].

### Pathway analysis of gene transcription data

Mouse transcriptomic data were analyzed by Gene Set Enrichment Analysis (GSEA version 2.07, www.broad.mit.edu/gsea) [21] using a gene set selected through the molecular signature database (MSigDB C3-MIR; www.broadinstitute.org/gsea/msigdb/collections.jsp#C3) for sharing 3′UTR sequences predicted to be targeted by miR-125a. Specific folders were created for each array and GSEA was performed for each transcriptome. Normalized enrichment score (ES) was calculated for each gene set. It reflects the degree to which a gene set is over-represented at the top or bottom of a ranked list of genes created by GSEA for each gene set according to differential gene expression between mice fed HFD or CHD. Positive and negative ES indicated that the gene set was overexpressed or underexpressed, respectively. For miR-125a associated gene set we selected the leading edge subset which is the subset of members of a gene set that contribute the most to the ES. A nominal p-value, estimating the statistical significance of the ES for a single gene set, was calculated after 1,000 permutations of the microarray samples to correct for gene set size and multiple testing, and a false discovery rate (FDR) was calculated. Gene set was considered significantly enriched when its p-value was less than 0.05 and FDR score was less than 0.25.

### Statistical analyses

Statistics were performed with Prism (GraphPad, version 6.0). Parametric student's t-tests were performed to compare means between two groups and ANOVA were performed to compare means between more than two groups.

Processing and analysis of the Affymetrix CEL file data was carried out using the BioConductor packages in the R language and environment as previously reported [6]. Gene chip data were normalised using RMA quantile normalization [22]. To correct for multiple testing, we used the FDR of Benjamini & Hochberg [23] to control the proportion of false positives at 5%.

## Results

### MiR-125a shows an opposite expression pattern in sensitive and resistant strains in response to high fat diet feeding

**Figure 1:**
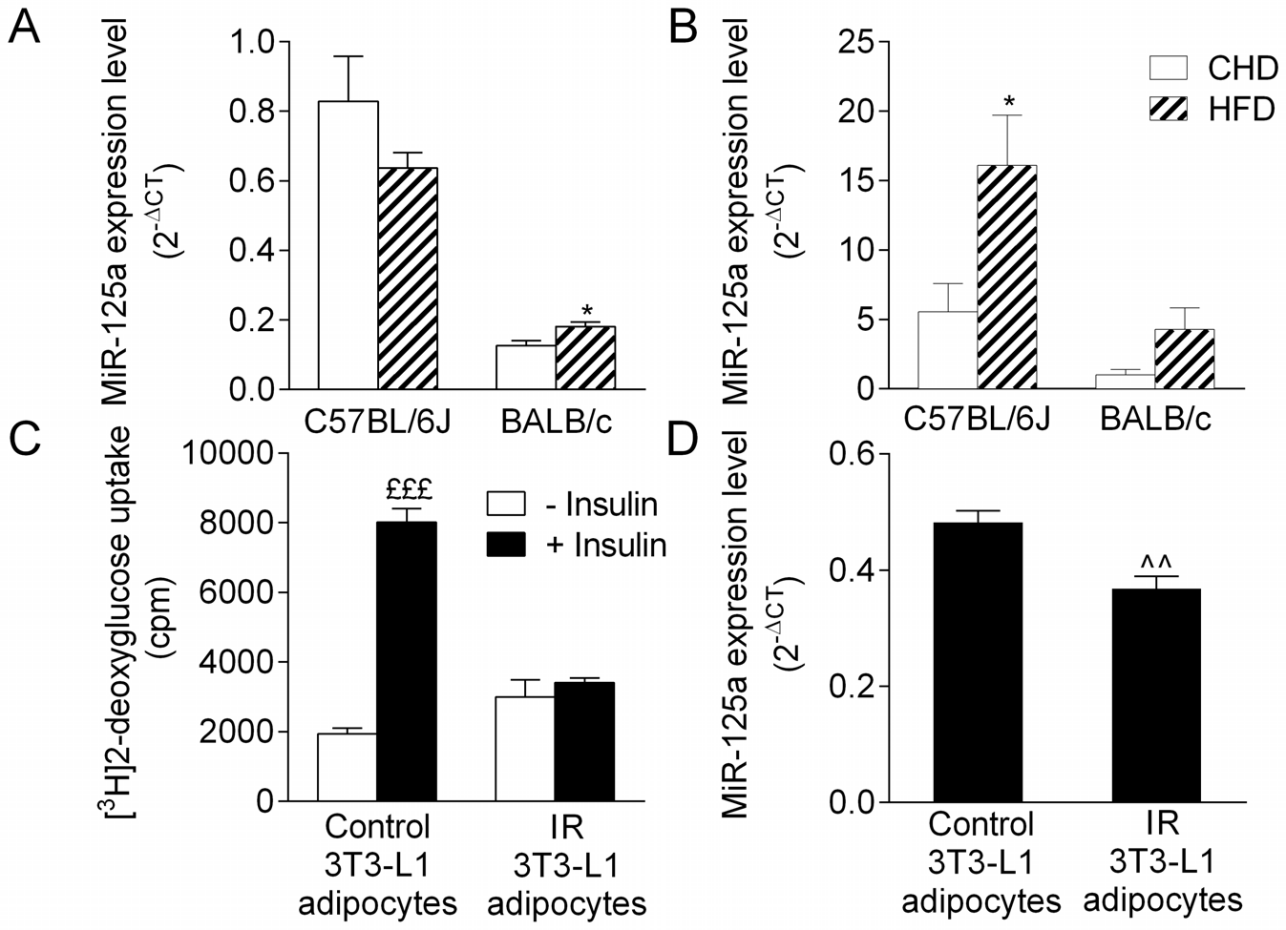
Biological relationships between insulin resistance and miR-125a expression. MiR-125a expression levels were quantified by q (quantitative) RT-PCR in adipose tissue of mice fed HFD for 1 week (A) or 15 weeks (B). The effect of *in vitro* insulin resistance (C) on miR-125a expression (D) was tested by qRT-PCR in 3T3-L1 adipocytes. Expression of miR-125a was normalized with small nucleolar (sno) RNA202 in adipose tissue and U6 in cultured cells. Data are expressed as mean ± SEM for 4–5 mice per group and for 3 replicated experiments in cultured cells. *p<0.05 significant differential expression of miR-125a between HFD-fed and control mice of the same strain. ^£££^p<0.001 significant differences in glucose transport stimulated by insulin in 3T3-L1 adipocytes. ∧∧p<0.01 significant differences in miR-125a expression between IR-3T3-L1 adipocytes and control-3T3-L1 adipocytes.

To test causal relationships between IR and miR-125a expression, we used complementary *ex vivo* and *in vitro* experimental systems designed to modulate either insulin sensitivity or the expression of this miR. We have characterized the short and long-term consequences of fat feeding on the onset and progression of diabetes and obesity in C57BL/6J and BALB/c (Table 2) [7], which demonstrate contrasting pathophysiological patterns and dynamics of adaptation to HFD feeding in the two strains. *Ex vivo*, in adipose tissue of BALB/c mice fed HFD for 1 week, miR-125a was significantly overexpressed when compared to CHD-fed mice (p<0.05) (Fig. 1A). This effect was not replicated in this strain after prolonged HFD feeding. In contrast, in C57BL/6J, miR-125a expression was unchanged in fat in response to 1 week HFD feeding but significantly overexpressed when the dietary stimulus was applied for 15 weeks (Fig. 1B).

**Figure 2:**
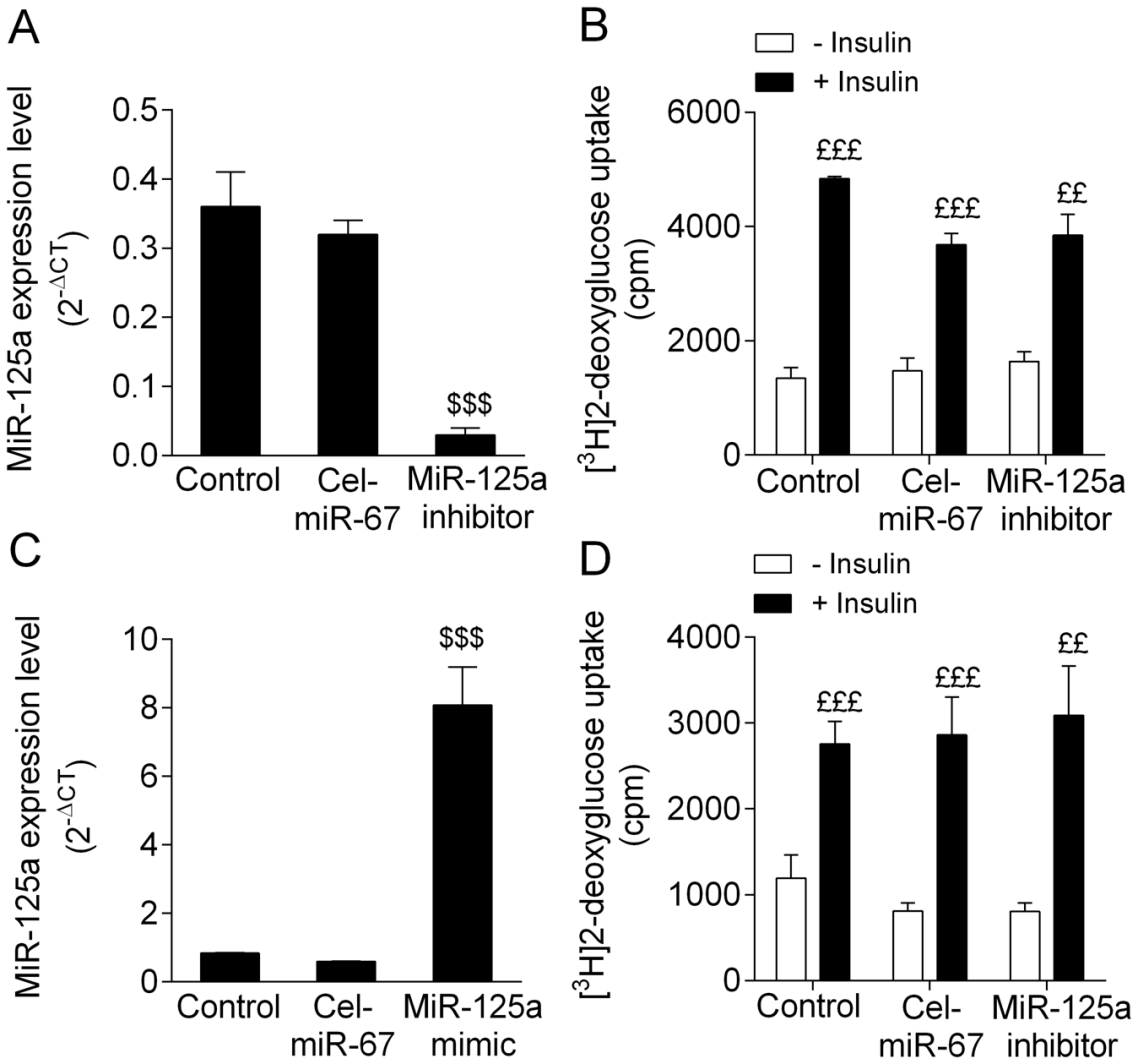
MiR-125a modulation of expression in 3T3-L1 differentiated adipocytes do not affect adipocyte glucose uptake. The effects of miR-125a expression downregulation (A) and upregulation (C) on glucose transport (B–D) were measured *in vitro* in 3T3-L1 differentiated adipocytes. Expression of miR-125a was normalized with U6. Data are expressed as mean ± SEM for 3 replicated experiments. $$$p<0.001 significant differences between miR-125a inhibitor/mimic, and control (not transfected cells) and cells transfected with C. elegans (Cel)-miR-67. £££p<0.001, ££p<0.01 significant differences in glucose transport stimulated by insulin in 3T3-L1 adipocytes.

We then tested the effects of miR-125a expression modulation on glucose transport *in vitro* in 3T3-L1 differentiated adipocytes (Fig. 2). We initially used 3T3-L1 differentiated adipocytes transfected with miR-125a inhibitor, which induced significant down-regulation of the expression of the miR by over 11-fold when compared to controls without inhibitor or with Cel-miR-67 (Fig. 2A). Our results showed that miR-125a inhibition had no significant effects on glucose uptake both in the basal state (without insulin) and after insulin stimulation (Fig. 2B).

MiR-125a was then overexpressed in 3T3-L1 adipocytes using a specific miR mimic. We obtained a 14-fold stimulation of miR-125a expression when transfected in the cells (Fig. 2C). Again in this system, miR-125a overexpression had no significant effect on glucose transport both in the basal state and after insulin stimulation (Fig. 2D).

Finally, we measured miR-125a expression in response to experimentally-induced IR in 3T3-L1 adipocytes (IR-3T3-L1) obtained after culture in a medium supplemented with high concentrations of glucose and insulin for 48 h (Fig. 1C–D). IR in IR-3T3-L1 adipocytes was characterized by quantification of insulin stimulated glucose uptake which was abolished in these cells (Fig. 1C). We demonstrated that miR-125a expression level was significantly decreased in these IR-3T3-L1 adipocytes when compared to controls (p<0.01) (Fig. 1D). Collectively, these results indicate that altered expression of miR-125a is secondary to IR *ex vivo* and *in vitro* rather than a cause of impairment of both glucose transport and insulin sensitivity *in vitro* in 3T3-L1 adipocytes.

### MiR-125a expression is downregulated in adipose tissue of obese patients

**Figure 3:**
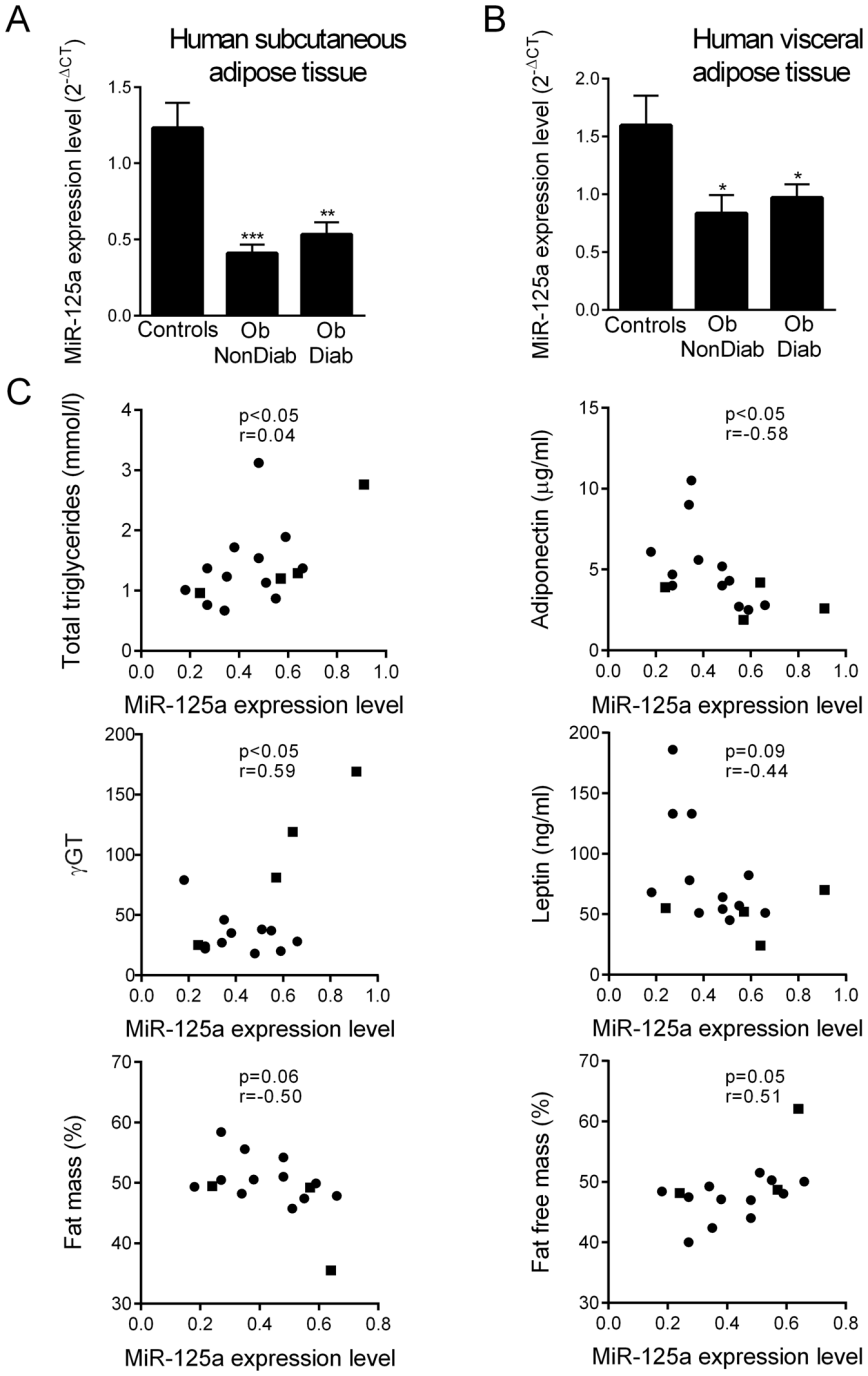
MiR-125a expression in adipose tissue of obese patients and correlations with physiological parameters. MiR-125a expression was quantified by qRT-PCR in subcutaneous (A) and visceral adipose tissue (B) in obese patients (Ob) with or without diabetes (Diab) and controls. MiR-125a expression level in obese patients was correlated with total triglycerides level, circulating leptin, γ-glutamyltransferase (γ-GT) and adiponectin levels, fat mass and fat free mass percentages (C) in males (▪) and females (•). Expression data were normalized with miR-16, an endogenous miR whom expression level do not vary across group of study. Data are the mean ± SEM of 6 non obese subjects for subcutaneous adipose tissue, 7 non obese subjects for visceral adipose tissue, 9 obese non diabetic and 7 obese diabetic patients. Parametric Pearson correlation tests were performed. *p<0.05, **p<0.01, ***p<0.001 significant differences between obese patients and controls.

To examine whether findings in rodent could be translated in human context, we quantified miR-125a abundance by qRT-PCR in biopsies of subcutaneous (Fig. 3A) and visceral (Fig. 3B) adipose tissue from obese patients with and without diabetes and from non-obese controls. We showed that miR-125a expression was significantly down-regulated in both subcutaneous and visceral fat of obese patients regardless of their diabetic status, when compared to lean subjects. To further document possible associations between miR-125a expression and intermediate phenotypes collected in obese patients, we calculated correlations between miR-125a levels and quantitative values of the phenotypes (Fig. 3C, phenotypes in Table 1). Expression level of miR-125a in subcutaneous adipose tissue negatively correlated with total fat mass (r = −0.50), serum leptin (r = −0.44) and adiponectin (p<0.05, r = −0.58), and positively correlated with fat-free mass (p = 0.05, r = 0.51) as well as with serum triglycerides (p<0.05, r = 0.04) and γGT (p<0.05, r = 0.59).

These results provide confirmatory evidence that miR-125a expression downregulation in adipose tissue is associated with obesity and underline its possible association with intermediate metabolic and hormonal phenotypes directly relevant to human obesity.

### Differential expression of miR-125a target genes is enriched in HFD-fed mice

**Figure 4:**
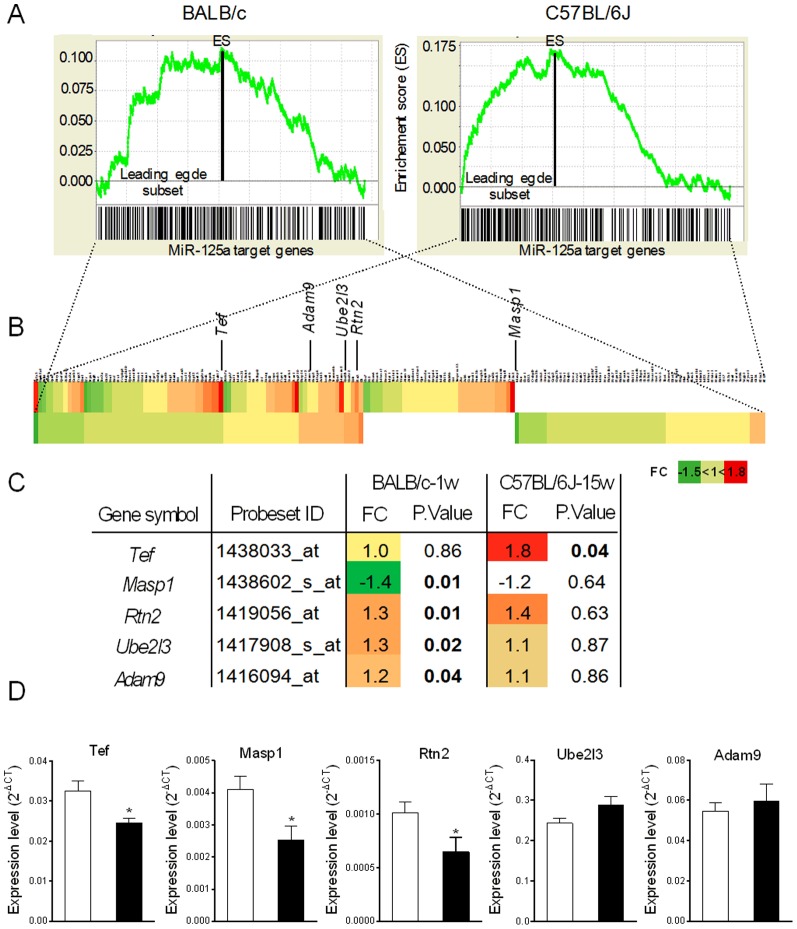
Expression patterns of miR-125a target gene sets in fat fed mice and in insulin resistant adipocytes. Gene set enrichment profiles of miR-125a gene targets (A) were determined for BALB/c mice after 1 week of HFD feeding (left) and C57BL/6J mice after 15 weeks of HFD feeding (right). Heatmap of miR-125a leading edge subsets are shown for fat fed C57BL/6J (top) and BALB/c (bottom) mice (B), alongwith enriched target genes significantly differentially expressed in these mice (C). Expression of *Tef*, *Masp1*, *Rtn2*, *Ube2l3* and *Adam9* was quantified by qRT-PCR in IR-3T3-L1 (▪) and control (□) adipocytes (D). Expression level was normalized with mouse *β-Actin*. Data are the mean ± SEM of 4 cultured cell replicates. *p<0.05 significant differences between IR-3T3-L1 adipocytes and control-3T3-L1 adipocytes.

To test the effects of miR-125a on expression patterns of its predicted transcript targets which can be further validated in human obesity, we carried out genome-wide gene expression studies in adipose tissue of mice previously used to demonstrate miR-125a differential expression in response to acute (BALB/c) or chronic (C57BL/6J) HFD feeding. Transcriptome data were initially analyzed globally with GSEA (see methods for details) using an inventory of groups of genes (gene sets) from the Molecular Signature Database C3-MIR that contains genes sharing a 3′-UTR binding motif of miR-125a, which are therefore likely transcript targets of the miR (Table S1). We identified a significant enrichment of miR-125a specific gene sets in the adipose tissue transcriptomes of C57BL/6J after 15 weeks of HFD feeding (p<0.001, FDR = 6.10^−4^) and BALB/c after 1 week of HFD feeding (p<0.02, FDR = 0.04). MiR-125a gene sets were overexpressed in both transcriptome datasets (ES = 0.17 for C57BL/6J and ES = 0.11 for BALB/c) (Fig. 4A). For each miR-125a associated gene set we selected the leading edge subset in order to retrieve the relevant transcriptome data (Fig. 4B and Table S1). Analysis of leading edge subsets in the two transcriptome datasets indicated that expression of 81 genes is upregulated and that of 30 genes is downregulated in C57BL/6J. Similar trends were found in BALB/c with 60 genes showing upregulated expression and 46 genes showing downregulated expression. Of note, GSEA highlighted largely different sets of enriched genes in the two strains (Fig. 4B, Table S1), which may result in different biological consequences.

Predominant overexpression of miR-125a targets in mice showing upregulated expression of this miR is counter intuitive with the general effect of miRs on gene transcription regulation and suggests its indirect role on the regulation of HFD induced transcription of the target gene set (Fig. 4A, B). We further investigated miR-125a mRNA targets (*Tef* (Thyrotroph embryonic factor), *Masp1* (Mannan-binding lectin serine peptidase 1), *Rtn2* (Reticulon 2), *Ube2l3* (Ubiquitin-conjugating enzyme E2L3), *Adam9* (Disintegrin and metalloprotease 9)) significantly differentially expressed in response to HFD feeding in the mouse transcriptomes. Expression of these genes was upregulated in response to HFD in C57BL/6J (*Tef*) or in BALB/c (*Rtn2*, *Ube2l3*). On the other hand, we observed significant down-regulation of the expression of the gene encoding *Masp1* in BALB/c, which is consistent with the expected transcriptional consequences of miR-125a upregulated expression in this strain. Our results suggest uncoupled regulations of the expression of miR-125a and its target genes in adipose tissue of fat fed mice, which may underlie pathological processes leading to obesity and IR.

To validate the coordinated regulation of the expression of miR-125a and its target genes, we investigated the transcription level of *Tef*, *Masp1*, *Rtn2*, *Ube2l3* and *Adam9* in IR-3T3-L1 adipocytes (Fig. 4D). The expression of *Tef*, *Masp1* and *Rtn2* was consistently significantly downregulated (p<0.05) in IR-3T3-L1 adipocytes when compared to control adipocytes, which cannot be explained by the downregulated expression of miR-125a in this experimental system.

### MiR-125a target genes are differentially expressed in adipose tissue of obese patients

Mouse-to-human translational studies of miR function rely on conservation of binding sites in target transcripts. We used Pictar (http://pictar.mdc-berlin.de/), which is an algorithm designed to identify microRNA targets based on 3′ UTR alignments, to verify that human Masp1, Rtn2, Tef and Ube2l3 also contain predicted binding site for miR-125a (http://pictar.mdcberlin.de/cgibin/PicTar_vertebrate.cgi?action=Search%20for%20targets%20of%20a%20miRNA&name2=hsa-miR-125a).

**Figure 5:**
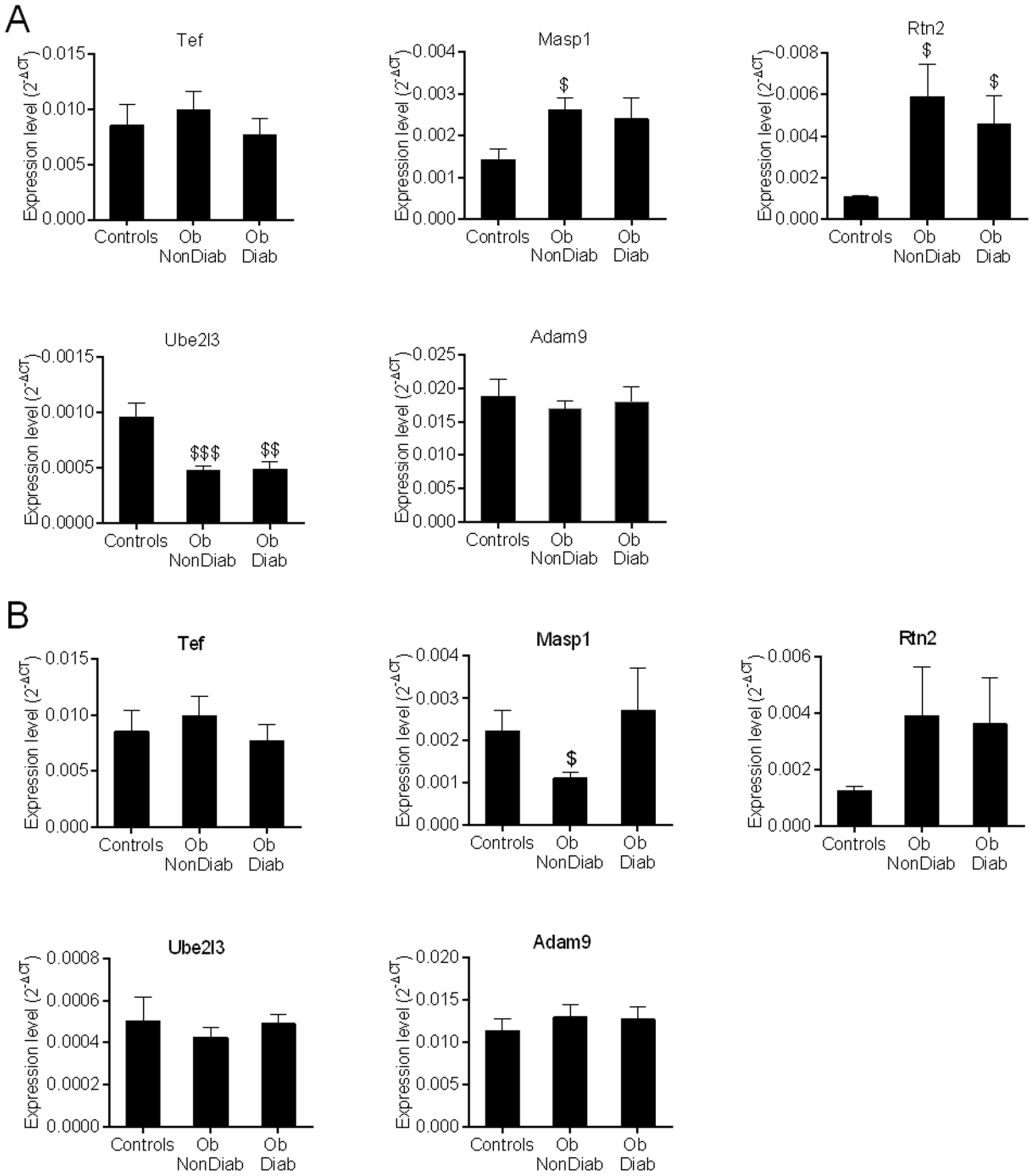
MiR-125a target gene expression regulation in adipose tissue of obese patients. Expression of *Tef*, *Masp1*, *Rtn2*, *Ube2l3* and *Adam9* was quantified by qRT-PCR in subcutaneous (A) and visceral (B) adipose tissue of obese patients (Ob) with diabetes (Diab) or without (NonDiab) and non obese subjects (Controls). Expression levels were normalized with human *β-Actin*. ^$^p<0.05, ^$$^p<0.01, ^$$$^p<0.001 significantly different to non obese subjects.

To test transcriptional effects of miR-125a down-regulation in human obesity, we quantified the expression of its target genes *Tef, Masp1, Rtn2, Ube2l3* and *Adam9* in subcutaneous adipose tissue samples of obese patients (Fig. 5A). *Tef* and *Adam9* were not differentially expressed between obese patients and non-obese controls. In contrast transcript levels of *Masp1* and *Rtn2* were significantly higher (p<0.05) in obese patients than in non-obese controls, regardless of the diabetic status of the patients, which may underlie a direct effect of miR-125a downregulated expression. Transcript levels of *Ube2l3* were significantly lower in subcutaneous adipose tissue of both obese non diabetic (p<0.001) and obese diabetic (p<0.01) patients which is inconsistent with the expected consequence of downregulated expression of miR-125a in obese patients, should *Ube2l3* be considered as an indirect target of this miR. In visceral adipose tissue, only *Masp1* expression level was significantly lower in obese non diabetic patients (p<0.05) than in non-obese controls (Fig. 5B). These results underline the incomplete concordance in expression patterns of miR-125a and its transcript targets in human obesity, which may indicate that these transcripts are indirect targets of miR-125a and may reflect altered molecular adaptations in obesity potentially contributing to disease pathophysiology.

### Target genes of miR-125a show contrasting expression patterns in adipose tissue of obese patients before and after gastric bypass surgery

**Figure 6:**
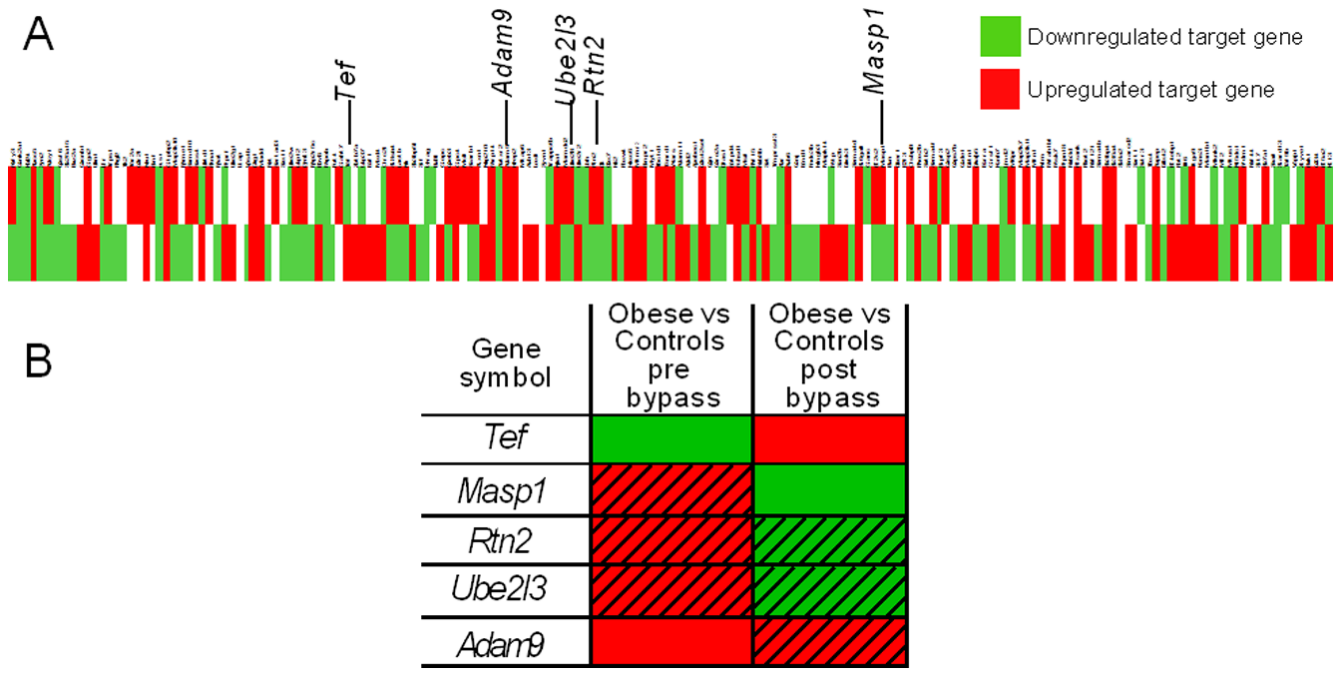
Expression profile of miR-125a target gene in adipose tissue of obese patients before and after gastric bypass. Heatmaps of miR-125a leading edge subsets are shown for obese patients before (top) or after gastric bypass (bottom) when compared to lean subjects (A). Patterns of expression for *Tef*, *Masp1*, *Rtn2*, *Ube2l3* and *Adam9* in these groups are illustrated. Cells are hatched when differences are not statistically significant (B).

**Figure 7:**
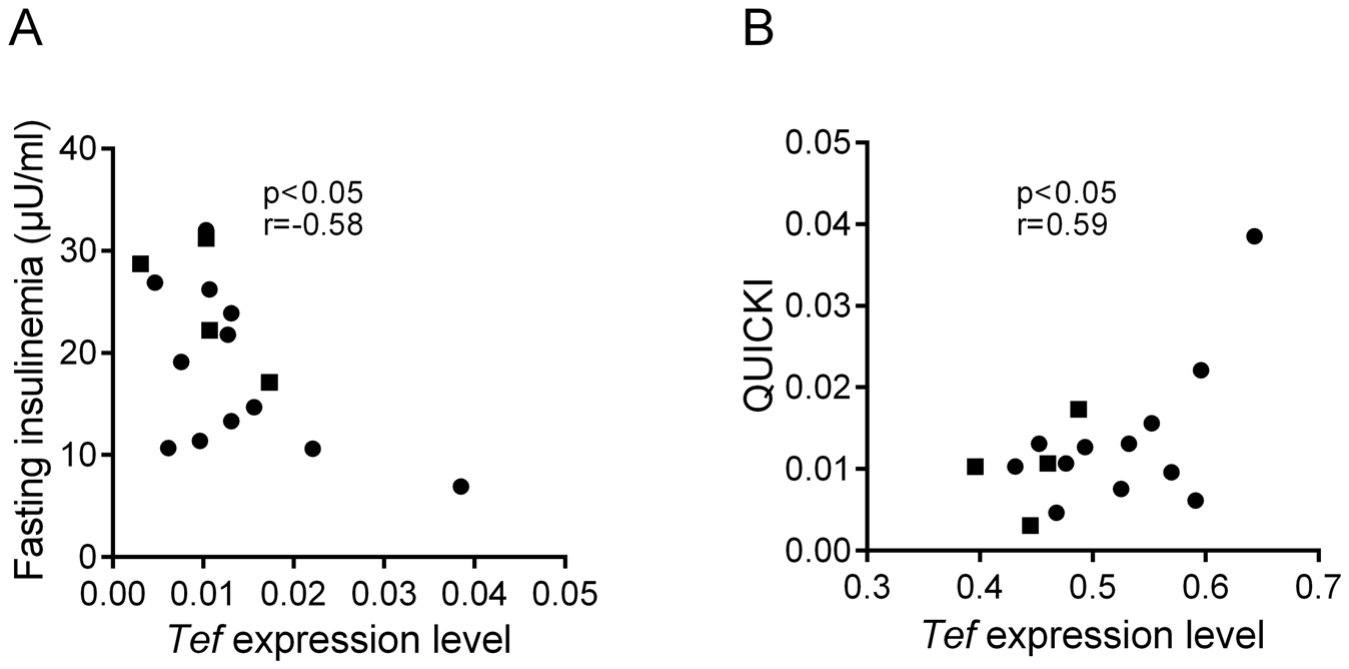
*Tef* expression level in subcutaneous adipose tissue is associated with fasting insulinemia and Quantitative insulin sensitivity index (QUICKI) in obese patients. Correlations between *Tef* expression level (2-ΔCT unit) and fasting insulinemia (A) and QUICKI (B) were calculated in male (▪) and female (•) obese patients.

Gastric bypass in severely obese patients induces weight loss and improved metabolic and inflammatory status [24]. We set out to investigate expression of miR-125a targets in obese patients before and after gastric bypass surgery. First, we analyzed the expression profiles of predicted miR-125a transcript targets, identified in GSEA leading edge subset, in obese patients before and after gastric bypass (Fig. 6A and Table S1). Expression profile analysis showed that 69 transcripts are downregulated and 39 transcripts are upregulated in obese patients when compared to non-obese controls. After gastric bypass more transcripts are differentially regulated in obese patients compared to control patients (80 are upregulated and 81 are downregulated). More specifically *Masp1*, *Tef*, *Rtn2* and *Ube2l3* showed contrasting expression patterns before and after gastric bypass (Fig. 6B) but the effects were statistically significant for *Tef* only, which highlights a possible specific role of *Tef* in obesity and IR of obese patients. We found a negative correlation between *Tef* expression level and fasting insulinemia (p<0.05, r = −0.58) and a positive correlation between *Tef* expression level and QUICKI, (p<0.05, r = 0.59) (Fig. 7). These findings further support the role of miR-125a and its transcript targets in the adaptation of IR and obesity in humans.

## Discussion

We report reactive expression of miR-125a and its predicted mRNA targets in adipose tissue from HFD-fed mice and obese patients. Expression patterns of this miR varied in response to an acute or chronic dietary stimulus in mice susceptible (C57BL/6J) or resistant (BALB/c) to diet induced obesity, as well as in insulin resistant adipocytes *in vitro*. Predicted transcript targets of the miR showed altered expression through GSEA of genome-wide transcriptome data in both C57BL/6J and BALB/c, but different subsets of genes contributed to global gene set enrichment in the two strains. Furthermore the expression level of miR-125a transcript target *Tef* was associated with insulin resistance status and *Tef* showed evidence of contrasting differential expression in obese patients before and after gastric surgery, a situation known to improve metabolic and inflammatory conditions. These results provide functional links between expression of miR-125a and its transcript targets in adipose tissue and IR and obesity.

MiR-125a shows a broad spectrum of functions, primarily characterized in development and cell differentiation, apoptosis, viral infection, carcinogenesis and autoimmune diseases [25], [26]. The impact of miR-125a in obesity, T2DM and IR, as well as in inflammation which is an important pathophysiological feature in obesity [27] relevant to our study have recently emerged from experimental studies in animal models and in humans. Abundance of miR-125a is increased in insulin resistant diabetic GK rats [14], as observed in fat fed mice in our study. Expression of miR-125a is also increased in macrophages after induction of proinflammatory response by oxidized LDL stimulation [28]. In humans, miR-125a expression is enhanced during adipogenic differentiation of multipotent stromal cells [29]. These observations support an acute physiological reactivity of miR-125a expression in adipose tissue of the genetically different mouse strains tested here, in a situation where adipocyte formation and inflammatory processes are stimulated by HFD-induced IR and obesity in mice showing strong susceptibility (C57BL/6J) and relative resistance (BALB/c) to these anomalies [7]. Conversely, downregulated expression of miR-125a in obese *ob/ob* mice [30], as observed in obese patients in our study, may underlie the existence of adaptative pathological mechanisms contributing to obesity pathogenesis in obese individuals.

A central issue in the possible involvement of miR-125a in IR and obesity lies in the definition of causal relationships between its abundance and phenotype alterations. We showed that modulation of miR-125a expression *in vitro* in 3T3-L1 adipocytes does not significantly affect glucose transport, whereas experimentally induced IR is associated with reduced miR-125a abundance. Our observations suggest that altered expression of miR-125a is reactive to IR and concur with results in obese patients. Correlations between miR-125a abundance and physiological parameters of insulin sensitivity in obese patients also suggest that changes in the expression of this miR reflect highly reactive adaptation to metabolic and/or hormonal changes associated with obesity, but may not play a direct causal role in the disease. Changes in miR-125a expression may have indirect pathophysiological consequences mediated by its transcript targets.

Identification of molecular mechanisms that mediate functional consequences of miR differential expression is a central issue in miR biology. Target genes of miRs share DNA elements that allow interactions between miRs and mRNAs, but the transcripts regulated by a miR do not necessarily share any biological function [31]. The definition of groups of transcripts regulated by miRs can provide crucial information to understand the biological endpoints of miR. However, it remains based on *in silico* prediction of base pairing interactions that requires experimental validation, for example through combined analysis of miR and gene expression, which allows direct integration of miRs in functional networks [32]. Parallel quantification of miR-125a and genome-wide gene transcription in adipose tissue in the same mice allowed analysis of coordinated expression of miR-125a and its predicted mRNA targets in response to HFD feeding. Following GSEA, we were able to establish a significant enrichment of miR-125a targets among differentially expressed genes in response to acute or chronic HFD feeding in BALB/c and C57BL/6J, respectively, thus providing experimental evidence that the genes encoding these transcripts are, to a large extent, genuine targets of miR-125a. These results also indicate that miR-125a expression reactivity to HFD, obesity and/or IR should have significant functional consequences in adipose tissue. Furthermore, transcripts contributing to gene set enrichment largely differ in C57BL/6J and BALB/c, which may account for metabolic and hormonal divergences in their response to HFD feeding.

We focused further analyses on five differentially expressed genes (*Adam9*, *Masp1*, *Rtn2*, *Tef*, *Ube2l3*) in the mouse transcriptome datasets, which we selected for their differential expression between HFD-fed and CHD-fed mice, their leading role in enrichment of miR-125a gene targets, and their known role in the regulation of body weight, insulin signaling or inflammation. *Adam9* is an insulin-like growth factor binding protein-5 protease associated with adiponectin, which may modulate the development of the metabolic syndrome [33]. Activation of *Masp1* can trigger local inflammation mediated by mannose-binding lectin binding to damaged endothelial cells [34]. The protein *Rtn2* plays an important role in membrane translocation of GLUT4 [35] and *Ube2l3* interacts with the ubiquitin ligase WWP1, a positive regulator of life span in response to dietary restriction in C. elegans [36]. Finally, *Tef* contributes to the circadian transcription of genes encoding acyl-CoA thioesterases leading to the release of fatty acids [37].

Based on expression patterns of the selection of miR-125a predicted targets, we did not find evidence of systematically conserved effects of changes in miR-125a level on transcript abundance in adipose tissue that may significantly contribute to obesity and IR in fat fed mice and in humans. For example, upregulated expression of *Masp1* in obese patients could reflect adipose tissue inflammation, whereas *Adam9* overexpression may underlie protective mechanisms against the metabolic syndrome. Consistent down-regulated expression of *Tef* in adipose tissue in C57BL/6J mice and in obese patients may underlie perturbations leading to IR and deteriorated lipid and glucose metabolism. Correlations between *Tef* transcript level, fasting insulinemia and QUICKI in obese patients support this hypothesis. Furthermore *Tef* showed contrasting expression patterns in obese patients before and after gastric bypass which may be due to improved insulin sensitivity, which occurs following gastric surgery [38].

An important challenge in miR studies in experimental systems remains to dissect out direct and indirect effects of miRs on gene expression and phenotypic endpoints. There is a general consensus supporting a predominant effect of miRs on gene expression silencing through various processes, even though examples of transcription stimulation have been reported [39]. One would therefore anticipate that expression of a miR and its mRNA targets should systematically exhibit coordinated regulation. However functional relationships between miR and mRNA are far more complex and miR abundance and activity can be regulated by the target mRNA it binds [31] These complex mechanisms may explain the general lack of coordinated regulation in the expression of miR-125a and its mRNA targets in fat fed mice and in obese patients. Nevertheless, contrasting expression of miR-125a and *Masp1* in both fat fed mice and obese individuals suggests that *Masp1* is a direct target of this miR, which mediates the effect of miR-125a on inflammation in the adipose tissue.

Collectively, our findings based on investigations of coordinated expression patterns of miR-125a and its mRNA targets in adipose tissue and cell lines underline the role of miRs in the biology of adipose tissue and contribute to improved knowledge of miR-125a mediated molecular mechanisms, bridging its known function in immunological processes [25] to novel molecular regulatory mechanisms relevant to IR and obesity pathogenesis in mice and humans. They suggest highly reactive expression of miR-125a in experimentally-induced and spontaneous IR and obesity, which may trigger molecular mechanisms associated with IR and inflammation in adipose tissue. Tissue level of miR-125a and some of its transcript targets may represent molecular markers of obesity and IR, as well as recovery processes from obesity and IR.

## Supporting Information

Table S1 **Mouse miR-125a predicted target gene expression profile in mice and humans are listed in table S1.**
(XLSX)
